# HEALTH, VITAL GOALS, AND CENTRAL HUMAN CAPABILITIES

**DOI:** 10.1111/j.1467-8519.2011.01953.x

**Published:** 2012-03-16

**Authors:** Sridhar Venkatapuram

**Keywords:** theory of health, capabilities approach, vital goals, Lennart Nordenfelt, Christopher Boorse, Martha Nussbaum, Amartya Sen

## Abstract

I argue for a conception of health as a person's ability to achieve or exercise a cluster of basic human activities. These basic activities are in turn specified through free-standing ethical reasoning about what constitutes a minimal conception of a human life with equal human dignity in the modern world. I arrive at this conception of health by closely following and modifying Lennart Nordenfelt's theory of health which presents health as the ability to achieve vital goals. Despite its strengths I transform Nordenfelt's argument in order to overcome three significant drawbacks. Nordenfelt makes vital goals relative to each community or context and significantly reflective of personal preferences. By doing so, Nordenfelt's conception of health faces problems with both socially relative concepts of health and subjectively defined wellbeing. Moreover, Nordenfelt does not ever explicitly specify a set of vital goals. The theory of health advanced here replaces Nordenfelt's (seemingly) empty set of preferences and society-relative vital goals with a human species-wide conception of basic vital goals, or ‘central human capabilities and functionings’. These central human capabilities come out of the capabilities approach (CA) now familiar in political philosophy and economics, and particularly reflect the work of Martha Nussbaum. As a result, the health of an individual should be understood as the ability to achieve a basic cluster of beings and doings—or having the overarching capability, a meta-capability, to achieve a set of central or vital inter-related capabilities and functionings.

## INTRODUCTION

Health and disease as well as related concepts such as illness, disability, impairment, and so forth have profound importance in modern societies. A range of rights and obligations often of great material and life-or-death significance flow from how these concepts are defined. Even at the supra-societal level, the concepts of health and disease are frequently used in evaluating the state of societies or to motivate global action. Moreover, it would seem prudent given the exponential rise in health development assistance as well as explosion in academic research programs on global health since the start of the new millennium to ensure or reaffirm that we have conceptual clarity on the concept of health.

The uncomfortable truth, of course, is that behind the billions of dollars of health development assistance, the multi-trillion dollar global healthcare industry, far reaching reorganization plans to improve public health, or public agitation for greater action on health inequalities and global health, we do not have a shared or coherent conception of health. If this assertion seems polemical and far-fetched, ask more than one individual whose work directly involves health if they could provide a definition of health. It will become clear quite quickly that the concept of health is used without much scrutiny. As Alan Cribb rightly describes it, health often seems to be ‘merely a useful compound label’ for a variety of things.^1^ So what then are we doing when we talk about health, healthcare, global health, or health equity and health justice?

The background or tacit understanding in the medical professions is that a person is healthy if they have no disease. And such a notion of disease is quite broad in that it encompasses infectious disease, chronic disease, injuries, poisonings, growth disorders, functional impairments and so on; disease encompasses all the conditions that are seen to be deviation from a ‘normal’ or ‘natural’ life course or physiological functioning of a human being. In public health, the aim is often to ‘contain and control’ diseases in populations that lead to impairments and mortality. In pursuing disease control, such policies are seen to be improving or protecting the public's health. And in health economics, the aim of economic evaluation or ‘cost-utility’ analysis of programmes is implicitly to maximize health. Health, in turn, is often defined by a mathematical function involving time, preferences (‘utility weights’), and levels of impairments from a disease condition. So the concept of disease plays a crucial role in our conception of health in a variety of important domains. At the least, disease means that health is not present, and health means that disease is not present. Improving health means controlling disease, and controlling disease means improving health. Nevertheless, the concept of health for many people does not only relate to the presence or absence of disease. It has to do with how they feel and what they are able to do. And even in the medical professions, given that some chronic diseases can be managed for decades, it seems inadequate to indefinitely categorize or label a person as unhealthy despite her successful management of a chronic disease.

The following discussion seeks to break the mutuality between disease and health. The theory of health I am advancing is not centrally moored to the concept of disease, and rejects the plausibility and pursuit of a value-free and scientific notion of health. Instead, I argue for a conception of health as a person's ability to achieve or exercise a cluster of basic human activities or capabilities. These basic activities are in turn specified through free-standing ethical reasoning about what constitutes a minimal conception of a human life with equal human dignity in the modern world. I arrive at this conception by closely following and modifying Lennart Nordenfelt's theory of health which presents health as the ability to achieve vital goals.^2^ Despite its strengths I transform Nordenfelt's argument in order to overcome what I consider to be three significant drawbacks. Nordenfelt makes vital goals relative to each community or context and significantly reflective of individual preferences. By doing so, Nordenfelt's conception of health faces problems with both socially relative concepts of health and subjectively defined wellbeing. Moreover, Nordenfelt does not ever explicitly specify a set of vital goals. The theory of health advanced here replaces Nordenfelt's (seemingly) empty set of preferences and society-relative vital goals with a human species-wide conception of basic vital goals, or ‘central human capabilities and functionings’. These central human capabilities come out of the capabilities approach (CA) now familiar in political philosophy and economics, and particularly reflect the work of Martha Nussbaum.^3^ As a result, the health of an individual should be understood as the ability to achieve a basic cluster of beings and doings – or having the overarching capability, a meta-capability, to achieve a set of basic inter-related capabilities and functionings.

The article proceeds as follows. The first section presents and reviews Nordenfelt's theory. And though I am enthusiastic about Nordenfelt's reasoning, I identify three weaknesses. I focus on the vagueness about vital goals, the standard circumstances clause, and the role of subjective preferences. Following that, I present my argument for conceiving health as the capability to achieve a basic set of capabilities and functionings. I then discuss some practical consequences and conclude the article.

## NORDENFELT'S THEORY OF HEALTH

Lennart Nordenfelt, a Swedish philosopher, has developed a theory of health partly as result of immense dissatisfaction with the prevailing view of health as the absence of disease and varied attempts to present it as a coherent theoretical concept. The most famous attempt to theorize health as the absence of disease was of course by Christopher Boorse.^4^ Boorse's theory of disease and health has profoundly shaped the parameters of the debates in the philosophy of health and medicine since the 1970s. The idea of disease as abnormal functioning and health as being a range of species typical functioning and involving a threshold (i.e. health is above the lower tail of normal distribution) has also influenced political philosophy.^5^ Despite its enormous influence and de facto status as the background theory of health and medicine, from its initial publication to the present it has provoked numerous and wide-ranging criticisms. Interestingly, in 1997 Boorse published his one and only rebuttal which sought to address over two decades of scrutiny.^6^ Nordenfelt published a counter-rebuttal in order to show that defects still remain in Boorse's theory despite the slight modifications.^7^ Though Nordenfelt may be less well known than Boorse, their respective theories have been identified, quite rightly I believe, as the two most important theories in the philosophy of health and medicine debates.^8^

Despite his thoroughgoing criticism of Boorse's theory, Nordenfelt sees his project as being very similar to Boorse's project. Nordenfelt also seeks to reconfigure or reconstruct and bring coherence to already existing health related concepts within healthcare as well as in everyday language. Nordenfelt's analysis starts with the commonplace idea that we think of health when it is not there; when there is instead, pain and disability. He then chooses disability as the primary concept rather than pain in proceeding to construct the definition of health because of the following reason. Even though pain can be due to disability, and pain can cause one to become disabled, all causes of pain are not necessarily due to disability. For example, heartache can cause pain, but it would be misguided to say that feeling pain from heartache is disabling and therefore, not healthy. The scope of pain exceeds disability, and does not always imply poor health. So grounding health or ill health in the concept of pain would lead us in the wrong direction. Focusing instead on disability, Nordenfelt then makes a linguistic move. He writes, ‘Disability is a negative notion presupposing the semantic content of its positive contrary, ability. This gives the analysis of ability a primary place in my theory of health’.^9^ He flips the focus from disability, which relates to the lack of health or being in ‘non-health’ onto the positive notion of ability, or where there is the presence of health. And then he asks, so what abilities should a healthy person have; what should a healthy person be able to do? In pursuing an answer Nordenfelt envisages human ability consisting of three parts: a human agent, their intended goal of action, and a supportive environment all of which come together to create the ‘real practical possibility of action’.^10^ This concept of practical possibility of action is informed further by the philosophy of action-theory, a disciplinary field which assesses ideas such as human action, causality, intent, basic action, and action-chains.^11^

In reflecting on what abilities a healthy person should have – what they are able to be and do – Nordenfelt focuses on the end goals of such abilities or actions; he calls these ‘vital goals’.^12^ In order to specify these goals, he considers and then rejects both ‘basic needs’ and satisfying desires. Basic needs are rejected because they are instrumental to other goals, or they presuppose a concept of health; some basic needs are derived from a concept of health. Desire satisfaction is also rejected because of harmful desires or low desires. Instead, he reasons that a vital goal of a person is ‘a state of affairs that is such that it is a necessary condition for the person's minimal happiness in the long run.’^13^ The stipulation of ‘in the long run’ is to avoid health being centred on immediate pleasure and instead, be more in line with long term happiness, thriving, or flourishing such as Aristotle's eudomonia. More recently, he rephrases the definition of vital goals as ‘a state of affairs which is either a component of or otherwise necessary for the person's living a minimally decent life. This includes more than survival’.^14^ Nevertheless, he does not fully discard satisfying desires or preferences. He reckons that a person's desires will still have a role to play even in Aristotelian happiness or flourishing. So what Nordenfelt tries to do is distinguish general desires or wants from the wants linked to life's most important or core goals. I shall return to this point about personal desires again further below.

In formal terms, Nordenfelt's theory of health is as follows:^15^ A is in health if, and only if, A has the ability, given standard circumstances, to realize his vital goals, i.e. the set of goals which are necessary and together sufficient for his minimal happiness.^16^

It is important to recognize, for reasons which will become clearer further below, that rather than talking about *abilities to act* in particular ways that aim to achieve a vital goal, for rhetorical ease he speaks directly about the vital goals. He clearly does not mean to say that health is reflected in levels of achievement of vital goals. Health is the *abilities* to achieve vital goals. Minimal happiness or flourishing is conceptualized as the outcome-states or achieving goals, but the health of a person is her abilities to achieve vital goals. Furthermore, though health is having the ability to achieve vital goals, to be unhealthy is not reflected by the not achieving of the vital goals, but by lack of the second order ability to acquire the first-order ability to achieve vital goals. Non-health is the lack of *capability* to produce the ability to achieve the goal. For example, either not being adequately nourished or not having the first-order ability to be nourished (e.g. feed oneself) is not enough to be labelled as being unhealthy. I am not healthy when I am not able to acquire or learn the ability to achieve adequate nutrition. If I am currently not well nourished but can become well nourished through learning or acquiring the ability to be well nourished, I am still healthy. The point at which I extinguish my own second order ability to do the first-order action, or some other biological event, another person, or an environmental factor destroys it, I then become ill or unhealthy.^17^ It is particularly insightful of Nordenfelt to account for such a second order ability. It recognizes that individuals move around different environments and/or require learning time to adapt, or some individuals may choose to not achieve their vital goals. Nordenfelt offers the example of an African farmer moving to a Nordic country. She may not immediately be able to achieve her vital goals in the new environment but after a period of time, she develops her abilities to achieve her vital goals. Looking at her achievements or first-order abilities might lead to concluding that she is unhealthy but it is her second-order ability that plays the pivotal role in her producing abilities and goal-achievements. The second order ability is defined as:

A has a second-order ability with regard to an action F, if and only if, A has the first-order ability to pursue a training-program after the completion of which A will have the first-order ability to do F.^18^

I would argue that Nordenfelt has brought us far in the path towards a coherent account of health. His corpus of writings as a whole show the inadequacy of Boorse's theory as well as express great care in systematically articulating his own theory while addressing a wide range of possible objections. Nordenfelt's methodological approach and architecture of the argument are very useful in conceptualizing health. But there are three weaknesses. I consider each in turn, and show how they can be overcome by integrating it with the social and political theory of human capabilities.

### Empty set of vital goals

The first weakness is that Nordenfelt stops short of filling in the framework of vital goals with any content. He explains why certain goals are vital but he does not identify the content of vital goals; what exactly are the vital goals that make a flourishing or minimally decent human life? Recently, Nordenfelt argued that vital goals across societies will have a similar ‘torso’. By that he means that across societies there will be some common body of content of the vital goals. He makes an analogy between the possibility of shared health concepts across extant societies with different notions of health over time within societies stating, ‘Health has always had to do with a person's well-being and ability related to his or her internal somatic and mental conditions.’^19^ Even if that is true, it is unclear why he stops short of articulating what is or could be the common body of vital goals within these general parameters. For example, even though he recognizes sheer survival or biological viability as only one of many vital goals of human beings, he does not specify this as part of the ‘torso’. This is at least one common vital goal across the entire human species, so should it not be specified in the theory? In response to such an assessment Nordenfelt could reply that he has in fact given consideration to the substance of vital goals. In a discussion in *On the Nature of Health* he writes, ‘Being alive is a necessary condition of being happy … Hence all the necessary conditions for maintenance of life must be included among every person's vital goals, for instance having food, having a sheltered home and having some economic security.’^20^ And so, from this discussion we are supposedly capable of deducing at least a shortlist of shared universal vital goals across the human species. While this may be a possible path, Nordenfelt himself has not in fact produced a short list of vital goals, and so his set of vital goals still remains (seemingly) empty.

### Standard circumstances

Nordenfelt is asserting, quite rightly, that health is not just a phenomenon internal to the body, something found within the biological structure, but also reflects the direct influence of the environment whether through physical or social forces upon the individual. When a person has no practical possibility to act because something or someone constrains their (second-order) capacity of action, then they are disabled and impaired, and not ‘in health’. However, the clause ‘given standard circumstances’ in his definition appears to be complete capitulation to the local social circumstances in determining the content of vital goals. This exposes Nordenfelt's theory to the charge of advocating social and ethical relativism. This is the second weakness of Nordenfelt's theory. While Nordenfelt may be reasoning about what a coherent understanding of health is, by defining it, he is also advocating how health should be envisaged. He may believe he is only making a logical point by describing the role of external conditions in a person's ability to achieve vital goals. For example, the standard circumstances usually have to include oxygen for a human being to achieve her vital goals. But because defining a concept has normative aspects, his definition cannot be seen to be adequate for its social, cultural, and ethical relativism. Which standard circumstances? Whose standard circumstances? Nordenfelt does recognize this tension as he states that local conditions can either be standard or reasonable. He suggests that in different discourses about health, we will either accept some notion of standard circumstances in assessing a person's abilities, and in others we will use benchmarks of reasonable circumstances.^21^ But it seems only to bolster further the point that the definition of health is contextually dependent on what is considered standard or reasonable for that scenario. And standard and reasonable are concepts with pivotal influence that come from outside his theory.

This possibility that health could be assessed against culturally relative circumstances or against what are considered to be reasonable circumstances, is unsatisfying. A theory of health is needed precisely to specify or evaluate rights and obligations related to health where local circumstances conflict with or fall well below what are considered to be reasonable circumstances. Assessing a person to be ‘healthy for their standard environment’ runs the risk of obscuring much ill health and injustice. One of the clearest illustrations of where common social practices and individual vital goals are not aligned is evinced in the high levels of endemic and acute mortality of girls and women in developing countries. Aside from biological vulnerability, the social, political, and economic practices that are locally determined undermine the abilities of girls and women to achieve basic functionings around the world. In particular, poor reproductive and sexual health in girls and women because of ‘standard’ patriarchal cultural norms leads to millions of avoidable deaths and impairments every year.^22^ Beyond the particular issue of women's health, the role of social arrangements in the causation and distribution of preventable mortality and disease is profound and pervasive across all human societies. Because the standard environment, or cultural norms can conflict with the achievement of vital goals of individuals, especially of those who are socially powerless, local cultural practices or the status quo should not have absolute determining power over the content of vital goals; or in determining who can achieve them, when, where, and how long. It is also important to recognize the influence, whether good or bad, of the social circumstances on how individuals identify and pursue their own vital goals. The choices we make depend on the choices we consciously or unconsciously think we have. The standard circumstance clause could potentially undermine the centrality of vital goals to health if the standard circumstances determine them. We want a conception of human health to be informed by but not wholly determined by local conditions and practices.

Nordenfelt takes us far by defining health as the abilities to achieve vital goals that lead to minimal happiness or minimally decent life. But he then suggests that these vital goals can be determined either locally or according to an external notion of ‘standard’ or ‘reasonable’ circumstances. In order for it to be a theory of health that covers the entire human species, the empty or socially relative definition of vital goals must be replaced with at least a core, stable, species-wide definition of vital goals. In searching for such a species-wide conception of minimal human welfare or wellbeing, there are a range of options. I could endeavour to identify a conception of vital goals based on my own personal conception of reasonable circumstances. However, it seems more prudent to draw on the large body of literature and state-of-the-art philosophical debates on human wellbeing or quality of life. In doing so, the overlap between Nordenfelt's vital goals and the idea of basic or central human capabilities, advocated by Amartya Sen and Martha Nussbaum, is striking. Nordenfelt's argument quite literally bridges the debates on the philosophy of health, medicine, and biology with the theory of human capabilities through the idea of health as the abilities to achieve vital goals. Nordenfelt's abilities to achieve vital goals are analogous if not the same as capabilities. The overlap should not be very surprising as upon closer examination it becomes clear that both Nordenfelt and the capabilities approach are informed by Aristotle's reasoning on action, influence of the environment, and human flourishing.^23^ Nordenfelt and Nussbaum also follow similar paths in identifying a conception of minimum human wellbeing or ‘happiness in the long run’.

## INTEGRATING VITAL GOALS AND CENTRAL CAPABILITIES

Nordenfelt is aware of the capabilities approach and has commented on relevant similarities and differences with Sen's arguments.^24^ He recognizes that his conception of health and wellbeing gives greater weight to subjective mental welfare, and that it is concerned with a smaller set of capabilities than Sen's general concern with human capabilities. Nordenfelt does not, however, consider Nussbaum's arguments for a set of basic capabilities. This would address his observation that capabilities theory is broader than his concerns about vital goals. The theory being advanced here is the integration of Nordenfelt's conception of health as the abilities to achieve vital goals with a list of core capabilities or freedoms. Thereby, health is having capabilities to achieve a certain cluster of capabilities and functionings. In this view health can also be seen as involving a second-order or over-arching meta-capability to achieve a cluster of basic capabilities and functionings. The justification for these capabilities being human species-wide, across all societies, comes from free-standing ethical reasoning about the importance of basic human freedoms and equal human dignity. Health is envisaged as fundamentally an ethical concept arising out of the values of human liberty and equal dignity.

Like Nordenfelt, Nussbaum also finds the basic needs and desire satisfaction approaches lacking in conceptualizing human well being.^25^ Though Nordenfelt was thinking about a person's health in relation to human flourishing and achieving vital goals, Nussbaum's main project is to define the components of a human life that reflect equal human dignity. Based on both Aristotle and Marx, she conceives human dignity as being able to be and do certain things; having certain capabilities. Through a method of dialectical reasoning very similar to Nordenfelt's, but asking what kind of life is worthy of human dignity – a minimally decent human life – across all societies, Nussbaum identifies such a life to consist of at least a threshold level of ten capabilities.^26^ And just like Nordenfelt, but for the different reason of valuing choice, Nussbaum also highlights the abilities and not the actual achievements. In contrast to Nordenfelt's reliance on ordinary language and action-theory philosophy, Nussbaum starts in the historical debates about natural law and sees certain ethical entitlements or claims implicit in the idea of human dignity.^27^ She then identifies a life worthy of human dignity to consist of ten capabilities including:

1) being able to live a normal length of lifespan; 2) having good health; 3) maintain bodily integrity; 4) being able to use senses, imagination, and think; 5) having emotions and emotional attachments; 6) possess practical reason to form a conception of the good; 7) have social affiliations that are meaningful and respectful; 8) express concern for other species; 9) able to play; and 10) have control over one's material and political environment.^28^

These ten capabilities, as moral entitlements emanating from a person's human dignity, become the source of political principles for liberal pluralistic society; ensuring each member achieves a threshold level of these ten central capabilities become primary political goals.^29^ So, unlike Nordenfelt who capitulates to the standard environment of various human societies or relies on the idea of reasonable circumstances external to his theory, Nussbaum defines what the standard environment should be in light of the moral, pre-political entitlements of human beings to the capabilities to achieve some ‘beings and doings’. But her conception also has room for individuals and different societies or countries to determine vital goals. Societies can add to the list of basic capabilities but cannot take capabilities away from the list, meaning that societies cannot choose to abdicate from supporting some capabilities. However, while the set of capabilities will be constant, different societies will determine the threshold levels of each capability depending on their history and resources. But these thresholds cannot be set only by domestic, ‘bounded’ reasoning or wholly determined by locally available circumstances. The levels must be determined through reasoning that involves a range of experts and stakeholders within and across societies.

In case there is any confusion, Nussbaum clearly considers her list of capabilities as constituting a conception of a life of minimal human dignity and not as providing a conception of health. But capabilities related to longevity and health are always listed first and second on her list of ten equally important capabilities. And even more intriguing is a footnote in *Women and Human Development*. In the footnote, Nussbaum writes that the definition of reproductive health adopted in the Section 7 of the Final Programme of Action of the 1994 Cairo Conference on Population and Development (ICPD) ‘fits well with the intuitive idea of truly human functioning that guides this list’.^30^ That is, Nussbaum appears to be saying that the definition of reproductive health as ‘a state of complete physical, mental and social well- being and not merely the absence of disease or infirmity, in all matters relating to the reproductive system and to its functions and processes’ fits with what she is trying to accomplish through specifying ten central human capabilities. This definition of reproductive health in turn mimics the World Health Organization's definition of health, which it should be noted, has frequently been disparaged as being nonsensical, utopian, and unfeasible. In any case, the implication of Nussbaum drawing a parallel between the ICPD/WHO's definition of health and the list of CHCs is that it is in fact possible to define health capability as being made up of all ten capabilities. And, the footnote shows that Nussbaum is aware of at least one account of health that could possibly encompass all ten CHCs. Moreover, Nussbaum's awareness that health could be conceived as being more than just the absence of disease seems to support the notion that the health capability on her list is really about the capability to avoid disease and impairments. Otherwise, she would be putting a list of capabilities within a health capability, and thereby, creating a list within a list. But why then does Nussbaum still use the label of health capability instead of referring to the capability to avoid disease and impairments? It may simply be the case that even here, the notion of health as the absence of disease has not been given sufficient scrutiny.

Indeed, if Nussbaum's list of capabilities proves to be troublesome as constituting health, we could develop a different set of basic capabilities. One could use alternative methodologies to come up with a different set of vital goals or capabilities, and it would still accomplish our goals of creating content for Nordenfelt's vital goals. For example, Gillian Brock argues that it is plausible to achieve global consensus on basic needs that are necessary for human agency; for ‘what a human being is like’.^31^ And Ingrid Robeyns has yet another method for identifying basic human capabilities.^32^ What is important is that the idea of health as the capability to achieve a cluster of basic capabilities and functionings still holds. Criticising one capability or functioning, such as the health capability on Nussbaum's list, does not undermine the argument that health should be seen as being able to achieve a cluster of basic capabilities or vital goals. To undermine that argument would entail going back and objecting to Nordenfelt's reasoning about health as being able to realize goals necessary and sufficient for minimal happiness; that is, a minimally flourishing, decent, non-humiliating life in the modern world.

### Vital goals too broad

Thomas Schramme offers one such objection to Nordenfelt's theory. This is the third weakness. He argues that the definition of vital goals is too broad particularly, as it relates to subjective preferences.^33^ Schramme illustrates his objection using the example of an ambitious athlete. Lily, a high jumper, has struggled for a long time to become an accomplished athlete, but has not succeeded and is not happy. Because Lily has not achieved her minimal happiness according to Nordenfelt's theory, she is not healthy. This seems odd to Schramme, especially as Lily has no disease. Moreover, Schramme also points out that by changing her goals, by becoming less ambitious, Lily could become healthy. Schramme's critique is that the inability of Lily to achieve minimal happiness because her ambitions outstripped her physical talents should not render her unhealthy. Nordenfelt replies that he views health as being on a spectrum from optimally healthy to maximal illness, presumably death. Therefore, Lily's unhappiness from not achieving her athletic goals moves her down on this scale away from optimum health. But she is unlikely to be ill/unhealthy unless her disappointment becomes debilitating. But for such a scale to work, Nordenfelt also needs to use thresholds to distinguish optimum from moderate health, and moderate health from poor or ill health. It is unclear where or how those thresholds will be created.

Some may find that Nordenfelt is letting a person's subjective preferences, or ideas of happiness, determine health too much. Consider the opposite scenario where a person who has a cheery disposition despite being impaired would be considered healthy, or moderately healthy. Or, a person with low ambition or low happiness thresholds would be considered healthy. While Nordenfelt understandably links vital goals to the emotional happiness of the individual, a strong role of subjective experiences of happiness determining health would make the conception incoherent. And it is not sufficient to say that ‘happiness in the long term’ will more closely resemble Aristotelian flourishing. There needs to be a greater specification of the extent to which emotional happiness or preference satisfaction will constitute the wider set of ‘long-run happiness’. However, if we use Nussbaum's list of capabilities as the content of Nordenfelt's vital goals, including the idea of sufficient thresholds, then Lily would be considered healthy. Rather than being considered unhealthy because she is emotionally unhappy due to not becoming a stellar athlete, if she is above the thresholds of basic capabilities, she would simply be an unhappy, disappointed person. She still has sufficient abilities to achieve her ten vital goals; health does not have to include the abilities to achieve any or all goals that will produce satisfaction or mental wellbeing. Nussbaum's list, by its breadth and sufficiency levels, allows for some level of subjective experiences but constrains the scope of vital goals from becoming total wellbeing. Thereby, it also constrains what is included in the conception of health. Health as a core set of capabilities represents a minimal conception of human wellbeing.

## PRACTICAL IMPLICATIONS

The theory presented here clearly moves the concept of health away from a central focus on disease or what is typical or most frequent functioning of internal biological parts and processes. It advances health as a person's capability to achieve or exercise some basic capabilities and functionings in the contemporary world. This obviously implies a cascade of consequences in a variety of health related domains. Foremost, reconfiguring our concept of health will affect epidemiology, the framework and methodology we currently use to study the causation and distribution of disease and mortality. Very few epidemiologists actually study the causation of health.^34^ Epidemiology and other health sciences would still continue to study the causes of diseases and impairments but they would also need to expand to study the causation and distribution of the cluster of basic capabilities and functionings. Conceptualizing and measuring capabilities is already being done in social exclusion studies, development economics, and in health economics.^35^ The natural sciences could provide knowledge about the biological bases of human capabilities. While the social sciences could provide knowledge about the social determinants of capabilities and their constraints. Furthermore, health policy will not just be confined to preventing and managing diseases but be more broadly concerned with protecting, promoting, sustaining, and restoring sufficient levels of capabilities to achieve functionings. It is not that surgeons will be expected to do more than surgery. But rather, what is considered to be health policy or health expertise will have to include more than healthcare. Health policy and expertise will have to encompass all the determinants of the different core human capabilities that constitute a minimally decent life.

This reconfigured conception of health will also profoundly affect how we respond to different distributions in health, the causes of the constraints on basic capabilities, their persistence, and the differential experiences or consequences of such constraints. And importantly, this ethical concept of health as the ability to achieve capabilities is a human species-wide conception; it puts the health of every human being across all societies on the same plane of observation and analysis. Health is defined in terms that reflect the equal dignity of human beings in the contemporary world.

If all this talk about health as achieving vital goals or cluster of capabilities and functionings seems too theoretical or fanciful, it should be noted that Nordenfelt's argument and my reconfiguration of it are really not radical or revolutionary. The criticism of Boorse's theory and the exhortation that we should be redirecting healthcare and health systems towards producing health rather than narrowly on preventing and managing disease has been a long-standing cause in the health sciences.^36^ Decades ago, Aaron Antonovsky even put forward a formal ‘salutogenic’ theoretical model of health to guide healthcare and health policy.^37^ And more recently, the World Health Assembly renamed the second edition of the International Classification of Impairments, Disabilities and Handicaps as the International Classification of Functioning, Disability and Health.^38^ This renaming reflects intellectual and social movements that seek to recognize health as being a spectrum from fully functioning in the world to being fully impaired, and explicitly recognizes that functionings or capabilities are co-produced by the features of the individual and surrounding environment.^39^

There has also been much work in health and medical sociology which examines how the concept of health and related social phenomenon are profoundly changing; they are becoming ‘diffused’ beyond a scientific or disease focus, beyond the individual body, and outside of the medical care system.^40^ All of this is to say that the concept of health is profoundly changing; it is not exclusively focused on the absence of disease, and is moving towards a holistic view.

Bringing together Nordenfelt's analysis with that of the capabilities approach has benefits for both. For Nordenfelt, his definition can become more defensible by incorporating the idea of basic human capabilities and justifiable through freestanding ethical reasoning. For the capabilities approach, Nordenfelt provides a link to the philosophy of health debates and offers a way of avoiding the pitfalls of using the notion of health as the absence of disease. Nussbaum faces exactly this problem with how she defines the health capability on her list. Also, the problems of ranking basic capabilities and the incoherent separation between the capability to live a long lifespan from the capability to be healthy get solved. Through health as a meta-capability, all the ten capabilities are recognized as being part of a cluster and inter-dependent.

Furthermore, some have argued that a non-scientific or ethical definition of health usually collapses into a conception of total wellbeing.^41^ Nordenfelt presents an argument for how health can be defined as a minimal account of wellbeing, or achieving vital goals. This argument combined with Nussbaum's reasoning about minimal conception of wellbeing helps to define health as minimal conception of human wellbeing. Nordenfelt provides the structure of health as the ability to achieve vital goals for minimal happiness that includes both subjective and objective content of vital goals, while Nussbaum provides the content of the vital goals in the form of ten central human capabilities. The breadth and extent of these basic capabilities reflect a conception of human dignity that encompasses the neediness, sociability and ability to reason in pursuing a life plan in the modern world.

## CONCLUSION

The main points to take away from this article is that health as a concept can be defensibly conceived as a meta-capability, the capability to achieve a cluster of basic capabilities to be and do things that reflect a life worthy of equal human dignity. Nordenfelt provides a line of reasoning to conceptualizing health as the ability to achieve a set of vital goals which, I have argued, is the same as saying having the capability to achieve a set of basic capabilities and functionings. While Nordenfelt arrives at his vital goals or minimally decent life through linguistic and action-theory philosophy, Nussbaum grounds her ‘central human capabilities’ in human dignity, equal respect, and other ethical values. And as I stated earlier, Nordenfelt and I are not the only ones seeking to define health in terms of abilities to function in the world. There are many other ways to get to health as a basic set of individual capabilities, thus bolstering an argument for seeing health as a meta-capability or ability to achieve a cluster of capabilities.

Finally, Nordenfelt's notion of health as the abilities to achieve vital goals really helps bring to the forefront the primacy of health in the lives of individuals and societies. If abilities to achieving vital goals are indeed the most important things in people's lives, then they (i.e. health) should be the most important social goals. Nordenfelt's reasoning when combined with Nussbaum's argument that the ten basic capabilities of citizens form the core of basic political principles, catapults health of citizens to the forefront of the social agenda. Philosophers and government officials often point out that health is only one among many pressing social goals, and individuals value health as only one among many other things in their life. Health-economists point to trade-offs that individuals are willing to make between health and other goods such as income. When health is properly understood as achieving vital goals, and the moral entitlements to the capabilities to achieve these vital goals are duly recognized as basic political principles grounded in freedom and equal dignity, the health of citizens becomes the first priority of social justice, and one of the most basic values of society.

## Footnotes

1 A. Cribb. 2005. *Health and the good society: setting healthcare ethics in social context*. Oxford: Clarendon Press: 22.

2 L. Nordenfelt. 1995. *On the nature of health: an action-theoretic approach*. 2nd revised & enlarged edn. Dordrecht: Kluwer Academic; L. Nordenfelt. 1987. *On the nature of health: an action-theoretic approach*. Dordrecht: D. Reidel; L. Nordenfelt et al. 2001. *Health, Science, and Ordinary Language*. Amsterdam: Rodopi; L. Nordenfelt. 2000. *Action, ability and health: essays in the philosophy of action and welfare*. Dordrecht: Kluwer Academic Publishers.

3 A. Sen. 2009. *The Idea of Justice*. London: Allen Lane: 468; M.C. Nussbaum. 2006. *Frontiers of justice: disability, nationality, species membership*. Cambridge, MA: The Belknap Press of Harvard University Press.

4 C. Boorse. On The Distinction Between Disease And Illness. *Philos Public Affa* 1975; 5: 49–68; C. Boorse. Origins Of Indeterminacy Thesis. *J Philos* 1975; 72: 369–387; C. Boorse. What A Theory Of Mental Health Should Be. *J Theory Soc Behav* 1976; 6: 61–84; C. Boorse. Wright On Functions. *Philos Rev* 1976; 85: 70–86; C. Boorse. Health As A Theoretical Concept. *Philos Sci* 1977; 44: 542–573.

5 N. Daniels. 1985. *Just health care*. Cambridge & New York: Cambridge University Press; J. Rawls. 1993. *Political liberalism*. New York: Columbia University Press: 182–185.

6 C. Boorse. 1997. A Rebuttal on Health. In *Biomedical ethics reviews What is Disease?* J.M. Humber & R.F. Almeder, eds. Clifton, N.J.: Humana Press: 1–134.

7 Nordenfelt et al., *op. cit*. note 2.

8 T. Schramme. A Qualified Defence of a Naturalist Theory of Health. *Med Health Care Philos* 2007; 10: 11–17.

9 Nordenfelt et al., *op. cit*. note 2, p. 67.

10 Nordenfelt 1995, *op. cit*., note 2, p. 41; L. Nordenfelt. On the notions of disability and handicap. *Int J Soc Welf* 1993; 2: 17–24., p. 17.

11 T. O'Connor & C. Sandis. 2010. *A companion to the philosophy of action*. Oxford: Wiley-Blackwell.

12 Nordenfelt, et al., *op. cit*. note 2, p. 67.

13 Nordenfelt 1995, *op. cit.* note 2, p. 93.

14 Nordenfelt et al., *op. cit*. note 2, p. 68.

15 To account for the health of human beings that do not have recognizable intentionality such as infants, he offers a modified theory. ‘Infant I is in health if, and only if, the internal constitution and development of I is such that, given standard adult support, the necessary and jointly sufficient conditions for I's minimal happiness are realized.’ Presumably, such a definition would also apply to individuals without full rationality or mental capacities. Nordenfelt 1995, *op. cit*. note 2.

16 Nordenfelt 1995, *op. cit*. note 2, p. 97.

17 In contrast to first and second-order abilities, I argue elsewhere that a human capability can be conceptualized as being constructed by the interaction of personal biological features, external social and physical conditions, and personal behaviours or agency. Furthermore, health is then reflected in the quality and breadth of a basic set of inter-dependent capabilities. S. Venkatapuram. 2011. *Health justice. An argument from the capabilities approach.* Cambridge: Polity Press.

18 Nordenfelt 1995, *op. cit*. note 2, p. 148.

19 L. Nordenfelt. Establishing a Middle-range Position in the Theory of Health: A Reply to my Critics. *Med Health Care Philos* 2007; 10: 29–32, p. 31.

20 Nordenfelt 1995, *op. cit*. note 2, p. 91.

21 Nordenfelt et al., *op. cit*. note 2, p. 68.

22 C.J.L. Murray & A. Lopez. 1996. *The global burden of disease : a comprehensive assessment of mortality and disability from diseases, injuries, and risk factors in 1990 and projected to 2020*. Boston, MA: Harvard School of Public Health on behalf of the World Health Organization and the World Bank.

23 M.C. Nussbaum. 1987. Nature, Function, and Capability: Aristotle on political distribution. In *World Institute for Development Economics Research Working Papers*. Helsinki; L. Nordenfelt & B.I.B. Lindahl. 1984. *Health, disease, and causal explanations in medicine*. Dordrecht: Kluwer Academic Publishers; Nordenfelt 1995, *op. cit*. note 2.

24 Nordenfelt 200, *op. cit*. note 2, pp. 94–105; personal communication

25 M.C. Nussbaum. 2000. *Women and human development: the capabilities approach*. Cambridge & New York: Cambridge University Press; M.C. Nussbaum & A.K. Sen. 1993. *The Quality of life*. New York: Clarendon Press.

26 Nussbaum, *op. cit*. note 25; Nussbaum, *op. cit*. note 3.

27 Nussbaum, *op. cit*. note 3, p. 37.

28 Ibid: 76–77; Nussbaum 2000, *op. cit*. note 25, pp. 78–80.

29 Nussbaum, *op. cit*. note 3, p. 70.

30 Nussbaum 2000, *op. cit*. note 25, footnote p. 83.

31 G. Brock. 2005. Needs and Global Justice. In *The philosophy of need*. S. Reader, ed. Cambridge: Cambridge University Press: 51–72.

32 I. Robeyns. Sen's Capability Approach and Gender iInequality: Selecting Relevant Capabilities. *Feminist Economics* 2003; 9: 61–92; I. Robeyns. Selecting Capabilities for Quality of Life Measurement. *Social Indicators Research* 2005; 74: 191–215.

33 Schramme, *op. cit*. note 8.

34 F.A. Huppert & N. Baylis. Well-being: Towards an Integration of Psychology, Neurobiology and Social Science. *Philos Trans R Soc Lond B Biol Sci* 2004; 359: 1447–1451; F.A. Huppert, et al. Introduction: Why Do We Need a Science of Well-being? *Philos Trans R Soc Lond B Biol Sci* 2004; 359: 1331–1332.

35 P. Anand et al. Capabilities and Well-being: Evidence Based on the Sen-Nussbaum Approach to Welfare. *Social Indicators Research* 2005; 74: 9–55; Ibid; J. Coast, et al. Valuing the ICECAP Capability Index for Older People. *Soc Sci Med* 2008; 67: 874–882; P.W. Saleeby. Applications of a Capability Approach to Disability and the International Classification of Functioning, Disability and Health (ICF) in Social Work Practice. *J Soc Work Disabil Rehabil* 2007; 6: 217–232, P. Anand. QALYs and Capabilities: a Comment on Cookson. *Health Economics* 2005; 14: 1283–1286; T. Burchardt. Capabilities and Disability: the Capabilities Framework and the Social Model of Disability. *Disabil Soc* 2004; 19: 735–751; E. Chiappero-Martinetti & J.M. Roche. 2009. Operationalization of the Capability Approach, from Theory to Practice: A Review of Techniques and Empirical Applications. In *Debating Global Society Reach and Limits of the Capability Approach*. E. Chiappero-Martinetti, ed. Milano: Fondazione Giangiacomo Feltrinelli: Grafica Sipiel.

36 I. Illich. Medical Nemesis. *Lancet* 1974; 1: 918–921..

37 A. Antonovsky. 1979. *Health, stress and coping*. San Francisco & London: Jossey-Bass.

38 World Health Organization. 2001. International Classification of Functioning, Disability and Health (ICF). Geneva: World Health Organization.

39 Saleeby, *op. cit*. note 35.

40 B.M. Turner. 2004. *The new medical sociology : social forms of health and illness*. New York: W.W. Norton; M. Blaxter. 2010. *Health*. 2nd edn. Cambridge: Polity.

41 D.W. Brock. 2002. The Separability of Health and Well-being. In *Summary measures of population health Concepts, ethics, measurement and applications*. C.J.L. Murray et al., eds. Geneva: World Health Organization.

